# High-temperature ultrafast ChipHPLC-MS

**DOI:** 10.1007/s00216-023-05092-w

**Published:** 2023-12-19

**Authors:** Chris Weise, Hannes Westphal, Rico Warias, Detlev Belder

**Affiliations:** Institute of Analytical Chemistry, University Leipzig, Linnéstrasse 3, 04103 Leipzig, Germany

**Keywords:** Chip chromatography, High-temperature chromatography, Microfluidics, Mass spectrometry

## Abstract

Herein, we present a miniaturized chip-based HPLC approach coupled to electrospray ionization mass spectrometry utilizing temperature to achieve high-speed separations. The approach benefits from the low thermal mass of the microfluidic chip and can form an electrospray from the pre-heated mobile phase. With the help of this technology, isothermal and temperature-programmable operations up to 130°C were pursued to perform reversed-phase separations of pesticides in methanol and ethanol-containing eluents in less than 20 s.

**Graphical Abstract:**

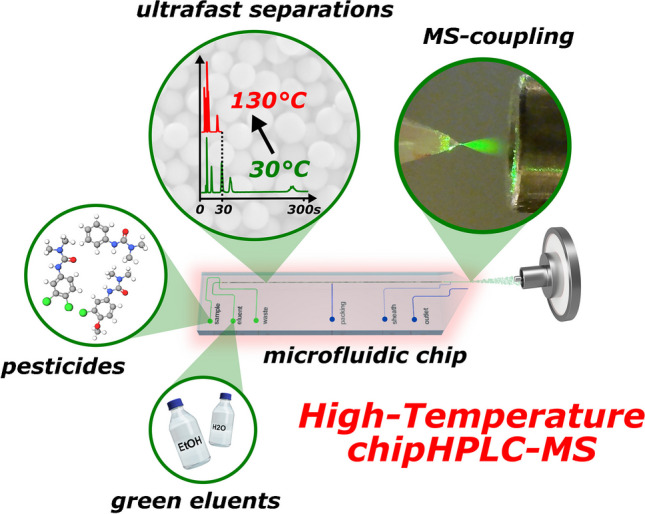

**Supplementary Information:**

The online version contains supplementary material available at 10.1007/s00216-023-05092-w.

## Introduction

Developing analytical systems that provide chemical data in ever shorter time frames is a current trend in chemical research. It can increase productivity and reduce operating costs, particularly in separation science [1–4]. Within this field, HPLC MS has established itself as the preferred method for qualitative and quantitative analysis of complex chemical samples. Although the combination of HPLC and MS is a powerful and high-resolution technique, its chromatographic front part is often time-consuming and leads to extended analysis times. Therefore, various approaches have been pursued to accelerate the analysis times from minutes to a few seconds, giving rise to the high-speed or ultrafast HPLC-MS field [5].

In addition to conventional approaches using sub-2-µm particles and increased pressures above 600 bars to accelerate HPLC, such as UHPLC, high temperature offers an exciting approach to get even faster [6–8]. The ability of high-temperature HPLC (HT-HPLC) to speed up chromatographic separation is based on the reduced viscosity of the employed mobile phase solvent and the increased mass transfer kinetics between stationary and mobile phase [9–12].

Another attractive feature of HT-HPLC is that the elution strength of the mobile phase increases with temperature so that gradient elution can be achieved by temperature programming similar to gas chromatography [13–16]. In addition, thermal equilibration of the column between runs occurs much faster than equilibration for solvent gradient and is another factor in accelerating HPLC cycle times. Since the speed of temperature adaptation scales with thermal mass, miniaturized systems such as the recently introduced high-temperature chip-based HPLC (HT-chipHPLC) are particularly promising when rapid heat exchange is required [17–19]. Despite the advantages that additionally result from the miniaturization of HT-HPLC, such as low microliter solvent consumption, absence of eluent pre-heating, and blazingly fast separations by thermal gradients, detector coupling in HTchipHPLC is challenging, as the column temperatures normally exceed the boiling point of the eluents used [20, 21]. This can lead to uncontrolled evaporation of the solvent before detection and cause signal instabilities [22]. As one of the remedial strategies to avoid the phase transition, the integration of back pressure stabilization in the post-column region of the HT HPLC microchip is proposed in the scientific literature. With this technology, the boiling point of the eluent is raised and the eluent remains in its liquid state.

Back pressure stabilization is convenient, especially with optical detection techniques, such as fluorescence, because conventional HPLC back pressure regulation equipment can be used without contributing to extra-column band broadening. Although maximum temperatures up to 180°C at backpressures of 69 bars can be reached briefly, undesirable temperature-dependent effects, such as signal quenching, limit fluorescence detection in HTchipHPLC [23].

In contrast to optical detection methods, coupling between HTchipHPLC and mass spectrometry is very appealing due to the higher information content. The electrospray process tolerates nanoliter per minute flow rates of heated eluents under ambient conditions. Although temperature alters ESI critical parameters, such as eluents surface tension and viscosity, numerous studies have emphasized the positive impact of temperatures up to 300°C during the electrospray process, leading to improved MS signal sensitivity without thermal degradation of the analyte [24–28].

Depending on the column’s inner diameter, interfacing strategies for HTLC MS are based on pre-detection-cooling and flow restriction [29–33]. As both approaches require additional post-column volume, either for sufficient heat exchange or for attachment of an external restrictive capillary, their use has a detrimental effect on separation performance. In this context, the lab-on-a-chip technology, with its unique feature of seamlessly integrating multiple functions onto a single chip device, can significantly improve by avoiding additional extra-column band broadening while providing the rapid heat exchange necessary to reduce the heated eluents temperature [34].

Various high-temperature chip chromatographs have been developed. However, a coupling between HTchipHPLC and MS remains vacant. Fueled by this motivation, the presented study aims to check the feasibility of MS coupling to HTchipHPLC. Therefore, we functionalized a glass-made microchip that utilizes the advantages of microfluidics to allow semi-automatically separate nanoliter sample fractions within a few seconds and achieve efficient MS detection of heated eluents using a monolithic chip emitter. Such a technology would push the boundary of analysis run times closer toward real-time analysis and broaden the analytical scope of HTchipHPLC by accessing the beauty of unambiguous identification through accurate mass measurements.

## Materials and methods

### Chemicals and reagents

Methanol, ethanol, isopropanol, acetonitrile, acetone, chloroform, and heptane were purchased in gradient grade purity (≥99.9%) from VWR International (USA). Formic acid in LCMS grade, purity ≥ 98%, was bought from Sigma-Aldrich (Germany). Sodium trifluoroacetate as MS calibrant in LCMS grade, purity ≥ 98%, was obtained from Sigma-Aldrich (Germany). 4-Amino-7-methylcoumarin (purity ≥99) from Sigma-Aldrich (Germany) was used for fluorescence experiments. Pesticide test samples composed of fenuron, cyanazine, diuron, fluometuron, metoxuron, tebuthiuron, and metolachlor, all PESTANAL® analytical standard purchased from Sigma-Aldrich (Germany). Thiourea (purity ≥99%) purchased from Sigma-Aldrich (Germany) served as a dead time marker. Chemicals for chip functionalization include H_2_SO_4_ (37%), acetic acid (98%) and 3-(trimethoxysilyl)propyl methacrylate (98%), 2,2-dimethoxy-2-phenylacetophenone (99%), trichloro(1H,1H,2H,2H-perfluorooctyl)silane (97%), 1,3-butanediol diacrylate (98%), and butyl acrylate (99%) all bought from Sigma-Aldrich (Germany). High-purity water (*σ*=18.2 MΩ cm) retrieved from Smart2Pure (TKA, Germany). PTFE cartridges filtered stock solution with a pore size of 0.22 µm, refrigerated at 4°C, and diluted to its desired concentration before use. XBridge C18 BEH particles with a particle diameter of 2.5 µm purchased from Waters (USA) were utilized as temperature-stabile stationary phase material.

### Microfluidic chip fabrication

The borosilicate (BOROFLOAT®33)-based microfluidic HT-HPLC chip (45 mm $\times$ 10 mm $\times$ 2.2 mm) has been manufactured by iXfactory, now part of Micronit (Germany), according to our design, and is illustrated in Fig. 2A. Chip fabrication was based on photolithographic, wet-etching, powder-blasting, and fusion-bonding techniques [35]. A microfluidic channel network is etched into the bottom slide during this process. The etched microfluidic channel network consists of a column structure (*l* 35 mm, *w* 90 µm, *d* 45 µm) with flow-restrictive weir structures (*l* 10 µm, *w* 45 µm, *d* 10 µm) on both ends. Furthermore, the column structure contains a packing channel diverting orthogonally halfway through the column and a microfluidic cross for sample injection and flow splitting. Peripheral accessibility to the microfluidic channel system was ensured by six conical-shaped connection openings integrated into the cover plate. The stationary phase was implemented by pressure-driven slurry packing temperature-stabile BEH C18 particles (dp=2.5 μm) via the packing channel [36]. The chip was submerged within a sonication bath to avoid particle aggregation of the slurry (1–3 mg·mL^−1^ prepared in ACN).

Porous monolithic frits were inserted at the beginning and end of the column before the packing process to retain the respective stationary phase material. After completing the slurry packing process, the packed column was sealed with a non-porous polymer. Both integrated polymers, the porous monolithic frits and the non-porous plug, are selectively introduced by LED-assisted radical polymerization.

For MS interfacing, a monolithic electrospray emitter was integrated into the microfluidic glass microchip. Therefore, the cuboidal-shaped front end of the glass microchip was ablated using a rotary grinder (Proxxon, Luxemburg). The resulting pyramidal-like shape served as an emitter tip. To increase contact angles and facilitate the electrospray formation of hydrophilic eluents, the monolithic emitter underwent silanization by dip-coating. After hydrophobization, the functionalized HT-HPLC microchip was installed into the chip thermostat and in front of  the MS orifice for measurement (Fig. 3F).

### Chip thermostat

The microcolumn thermostat’s components and basic functionality were described previously [19]. In brief, two cylindrical micro thermoelements (20 W, *t*_max_=260°C) embedded into a polyether ether ketone (PEEK) housing, driven by a 24 V DC power supply, served as infinite heat sources. Temperature surveillance by three Pt100 provides a sensing element for an integrated PID loop controlled by custom-built LabVIEW software. HPLC chip and chip thermostat are interfaced by a clamp fixture to directly contact-heat to the microcolumn both-sided up to 200°C with a rate of 4.7°C/s. Before the chromatographic operation, the microcolumn was thermally equilibrated for 60 s. Infra-red radiometric imaging was conducted using a FLIR PRO One (USA) mobile camera.

### Sample injection and elution

Sample injection onto the packed column is realized by an adopted hydrodynamic pinched injection scheme as described earlier [37]. Briefly, this is realized by two pump-driven fluidic situations, referred to as injection and elution.

An overview of both fluidic situations, injection and elution, is provided in the Electronic Supplementary Material Fig. S1 and S2. Switching from injection to elution mode is done via two external 10-port nano switching valves (Cheminert, 100 µm bore size, VICI, Switzerland), of which the first nano valve is equipped with a 4.6-μL sample loop to forward a sample plug to the second nano valve. The second nano valve selectively directs the sample and pinch stream or elution stream towards the injection cross on the microchip. During the sample injection, a partial volume of the sample plug is loaded onto the column head and remains there until its elution. During the study, the injection mode was maintained for 15 s before switching to elution mode. During elution, the eluent pump flow increases the pressure at the injection cross and flushes the sample plug from the column head along the column. The entire process was executed by a semi-automated injection protocol using Clarity Software Package (Data Apex, Czech Republic).

### Mass spectrometric detection

A high-resolving quadrupole-orthogonal time-of-flight instrument (micrOTOF-Q II, Bruker Daltonics, Germany) was used for mass spectrometric detection.

A home-built chip interface allowed the micro-thermostat, two 10-port nano valves, and a pressure sensor (Duratec, Germany) to be securely affixed near the associated microchip. An XYZ-axis stage micromanipulator (Thorlabs, USA) ensured precise spatial positioning in front of the inlet of the mass spectrometer, whose inlet capillary extension was customized for chip interfacing. For chromatographic experiments, all mass spectra were acquired with up to 4–8 Hz in full scan mode (mass range 100–415 m/z). At the MS inlet, a potential between 2.5 and 3.5 kV was applied. Low dry gas flows of 1 L/min at 225°C were used during operation. Ion chromatograms and mass spectra were analyzed with Data Analysis 4.3 (Bruker Daltonics, Germany).

## Results and discussion

The main focus of the study is to develop a technical approach to interface between high-temperature chipHPLC and ESI mass spectrometry. By increasing the column temperature, the increased eluotropic strength of the eluent and enhanced mass transfer kinetics should be exploited to shorten the analysis time of the chip-based chromatography. This objective raises the question of the temperature limits within which electrospray formation on the heated chip emitter is possible. In a previous HTchipHPLC study, the chip outlet was connected to an external HPLC backpressure regulator to prevent evaporation of the mobile phase in the microchip through backpressure stabilization [19]. Assuming that only a minor part of the microchip, containing the packed column, is heated and the emitter is located in the non-heated part, trouble-free electrospray operation could be possible due to the rapid heat exchange and pressure drop within the microchip. This was investigated in a preliminary experiment by dosing a sample plug (20 nL of 150 µM metolachlor dissolved in MeOH) onto a heated soda-lime glass test chip with subsequent MS analysis.

The home-built soda-lime glass test chip contained a single micro-structured channel and was designed to heat parts of the channel when placing the chip into the microchip heater. For MS interfacing, an emitter was integrated on-chip by cutting and grinding off glass material in the front of the test chip to form a monolithic emitter in a pyramidal shape. The emitter tip underwent hydrophobization to prevent spray instabilities caused by low contact angles. The final soda-lime glass test chip was connected to a nano injection valve (Cheminert, VICI, Switzerland) to allow injection of a small nanoliter injection volume into the 1 µL/min eluent stream consisting of 70:30 v/v MeOH:H_2_O, 0.1% FA. Due to the fluidic connection between the external injection valve and the test chip, the chip operated at ground potential and could, therefore, form an electrospray between the emitter tip and the high voltage MS inlet to detect protonated metolachlor ions, [M+H]+, under ambient conditions.

Since this preliminary experiment aimed to investigate the MS performance at higher temperatures, the chip thermostat gradually heated the soda-lime glass test chip from 30 to 180°C. At each temperature step, a nanoliter sample plug of metolachlor was injected onto the heated chip, and its extracted ion chromatogram (EIC) at 284 m/z was detected. The recorded data indicate that it is possible to record ESI-MS signals of metolachlor for temperatures up to 180°C. Furthermore, the detected maximum intensities of the metolachlor ion chromatograms increased to temperatures of 130°C.

Since phase separation is observed when working at high temperatures, it was unexpected that electrospray formation could be maintained so far above the boiling point of the solvent (bp =71°C) in a microfluidic channel that provides a back pressure of 1 bar under the given conditions. The observed effect of increasing ESI-MS sensitivity at elevated temperatures is consistent with work done on ESI-MS using dedicated ESI sources with heated nitrogen or inlet capillaries [24, 27, 28].

More information about the preliminary investigation, including an illustration of the soda-lime test chip, a detailed experimental setup, and the recorded MS data, can be seen in the Electronic Supplementary Material Fig. S3.

**Fig. 1:**
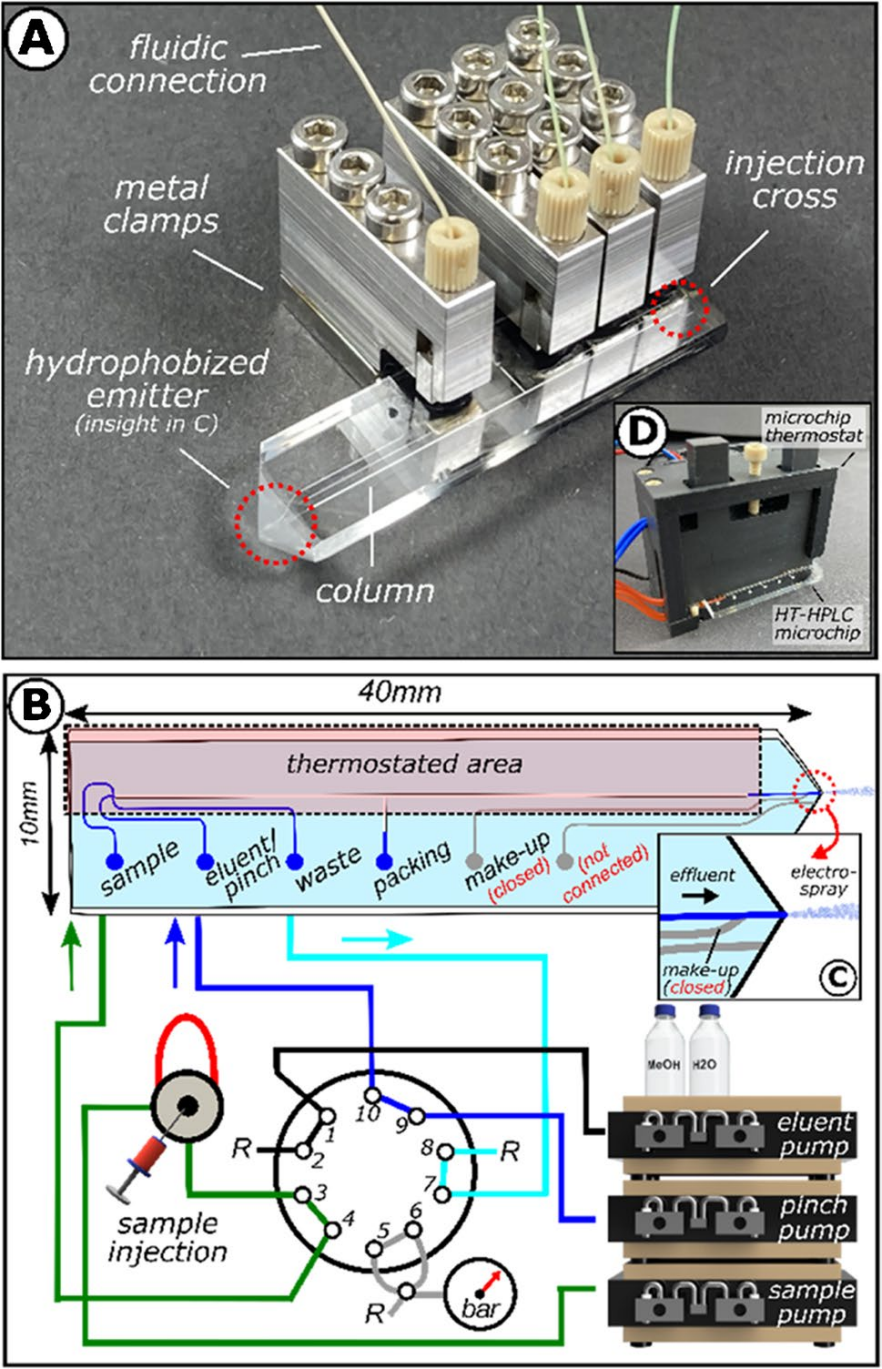
Overview of the microchip design and experimental setup of the HTchipHPLC-MS approach. **A** Photographic image of the functionalized HT-HPLC microchip. **B** Schematic drawing of the external fluidic circuitry in injection mode. It includes the microfluidic design of the microchip (top view) and fluidic components, such as HPLC pumps, valves, and a pressure sensor. The flow paths of the sample stream (green), pinch stream (blue), and eluent stream (black) are colored. Since the sample and pinch streams leave the chip at the waste outlet to flow into one of the restriction coils (*R*), the corresponding tubing is colored cyan. Capillary tubings not connected to a pumping device in this fluidic situation are colored in gray. In addition, arrows indicate the direction of flow. **C** Insight of the post-column layout of the HT-microchip. **D** Photographic image of the microchip thermostat with the functionalized HT-HPLC microchip installed. More details about the experimental setup used in the presented study, including the fluidic circuitry for elution mode, visual inspections of the microfluidic cross injecting a fluorescent sample, and pressure data, are given in the Electronic Supplementary Material Fig. S1 and S2

After these encouraging first experiments with a simple partially heated test chip with a non-restrictive channel and an emitter tip, we developed a functional HT-chip HPLC. A photographic image of the HT-HPLC microchip and a general overview of the experimental fluidic setup used in this HTchipHPLC approach are shown in Fig. 1A and B.

The layout of the developed HT-HPLC microchip consisted of an injection cross, a 35-mm-long HPLC column packed with temperature-stabile C18 BEH particles. In contrast to the previous HTchipHPLC approach, there is now an electrospray emitter at the end of the chip (4 mm from the column end) instead of the capillary attached to a conventional HPLC backpressure regulator. The electrospray emitter, shown in Fig. 1C, was manufactured by grinding and made hydrophobic by silanization [35].

The HT-HPLC microchip was connected to an external fluidic circuitry via home-built steel clamps to ensure operation [38]. The connecting clamps provide a pressure-stable connection even at 200°C. With regard to high-temperature compatibility, flow restrictions in the pre-column region were adapted to apply the pressure necessary to avoid phase separation of the heated eluents.

**Fig. 2:**
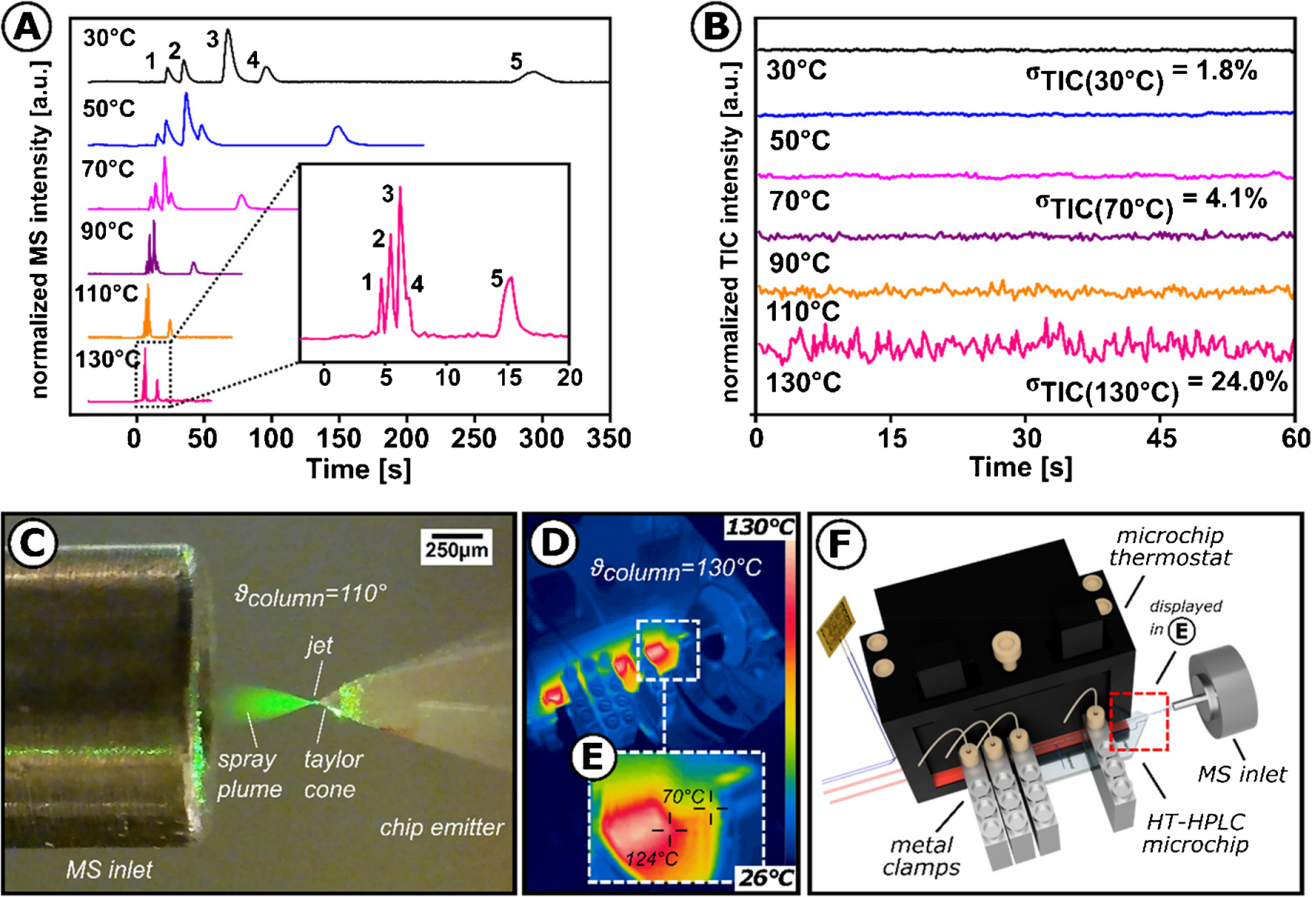
**A** HTchipHPLC under isothermal conditions with MS detection (from *ϑ*_µ-column_ =30°C to 130°C, whereas *ϑ*_column_ =130°C is displayed within insight view), column length: 35 mm, material: XBridge C18 BEH, dp=2.5 µm, maximal elution pressure: 132 bar, pesticide mixture containing fenuron (1, *c*=50 µM), cyanazine (2, *c*=50 µM), diuron (3, *c*=50 µM), fluometuron (4, *c*=50 µM), and metolachlor (5, *c*=50 µM) dissolved in 50/50 v/v MeOH/H_2_O, 0.1% FA was injected. All phenyl urea pesticides were detected as [M+H]^+^ can be found in Electronic Supplementary Material Fig. S4. **B** Stability of the MS baseline signal under isothermal conditions (listed from *ϑ*_column_ =30°C to 130°C), intensity of the normalized total ion current (nTIC) and its corresponding deviation depicted as *σ*_TIC(ϑµ-column)_ in % are displayed, no sample injected, 50/50 v/v MeOH:H_2_O, 0.1% FA was used as an eluent. **C** Electrospray formation within the developed HTchipHPLC MS interface using heated eluent (50:50 v/v MeOH/H_2_O, 0.1%, *ϑ*_column_ =110°C, MS inlet: 3.5 kV). **D** Thermographic image of the HTchipHPLC assembly at column temperatures of 130°C. **E** Insight of the temperature distribution in the post-column area of the microchip. A linear IR-radiation scale and an emissivity of *ε*=0.88 for the microchip were applied. **F** Illustration of the HTchipHPLC MS assembly installed in front of the mass spectrometer. The red dashed box points out the post-column region of the assembly.

For temperature control, the HT-HPLC microchip assembly was installed to the microchip heater (Fig. 1D), securely affixed to the XYZ micromanipulator, and positioned in front of the aperture of a mass spectrometer. An electrospray could be formed after precise alignment with the MS inlet while the microchip was held at ambient temperature. The developed assembly has been evaluated by injecting a sample mixture of five phenyl urea pesticides. To this end, optimizing the eluent composition at a constant column temperature was necessary. Here, an isocratic solvent composition of 50:50 v/v MeOH:H_2_O, 0.1% FA at 30°C at an elution pressure of 132 bar was found to baseline separate all five analyte compounds, as illustrated in the top part of Fig. 2A. Therefore, this elution composition was used for further chromatographic experiments.

After the eluent optimization was completed, experiments were carried out to investigate the performance of the developed chipHPLC-MS setup under high-temperature conditions. For this purpose, the column temperature was increased stepwise without sample injection. During the temperature increase, electrospray formation and the behavior of the total ion current (TIC) was observed.

Using an MS inlet voltage of 3.5 kV, the developed HTchipHPLC MS system could generate an MS ion signal for a column temperature of 110°C.

The microscopic examination confirmed the formation of an electrospray consisting of a cone, a jet, and a spray plume between the emitter tip and the MS inlet (Fig. 2C). Since the cone formation requires a liquid state, it can be assumed that the heated eluent in the post-column area is subject to a strong heat exchange. In the case of the used eluent consisting of 50:50 v/v MeOH:H_2_O, 0.1% FA, a heat exchange below its boiling point of 76°C is therefore assumed. Furthermore, it was found that the fluctuations of the TIC increase with increasing column temperature. For example, the relative standard deviation of the 1-min TIC acquisition is 1.8% at 30°C and rises to as much as 24% at 130°C (Fig. 2B). To investigate possible causes of the TIC fluctuations, the temperature distribution over the entire microchip operating at 130°C was visualized by thermographic imaging using a mobile thermal imaging camera (Fig. 2D). For a better orientation, a true-scale illustration of the HTchipHPLC arrangement in front of the MS inlet is displayed in Fig. 2F.

The thermographic image illustrates that surface temperatures of areas in direct contact with microchip thermostat correlate to the set column temperature. In contrast, areas at the chip edges generally have lower surface temperatures, indicating the rapid heat transfer of the microchip. Exemplarily, the surface temperature in the post-column region was lowered from 124°C at the end of the column to 70°C at the emitter tip (Fig. 2E).

This heat exchange was sufficient to reduce the surface temperature below the boiling temperature of the eluent. Realizing an even larger temperature difference in the post-column region remains a challenge since the borosilicate glass substrate cannot dissipate the applied heat fast enough. More efficient heat dissipation should be possible by active means, e.g., integrated microfluidic cooling channels or a cooling gas flow directed to the ESI tip.

In the presented setup, gas bubbles were observed to elute from the column into the post-column region of the HT-HPLC chip at too-high temperatures.

Since phase transition can be prevented by raising the post-column pressure, the ability of the post-column channel (trapezoidal cross-section, *l* 4 mm, *w* 70 µm, *d* 30 µm) to build up sufficient backpressure was questioned. Subsequent estimates using the Hagen-Poiseuille equation (viscosity_(50:50 MeOH:H2O)_ = 1.34 cP, total elution flow rate = 80 µL/min) show that the pressure drop in the post-column channel is in the millibar range, which explains the presence of gas bubbles in the post-column region of the chip and the increasing fluctuations of the TIC. Nevertheless, it is remarkable that the TIC oscillates constantly, even with more significant fluctuations, and never breaks off entirely up to a column temperature of 130°C. This indicates a certain tolerance towards an outgassing eluent [39].

In the following set of experiments, injections of the pesticide mixture were made at increasing column temperatures from 30 to 130°C. A 60-s equilibration period was implemented before each new temperature step to avoid undesired temperature gradients. A plot of the recorded isothermal separations with ascending column temperature from top to bottom is shown in Fig. 2A.

**Fig. 3:**
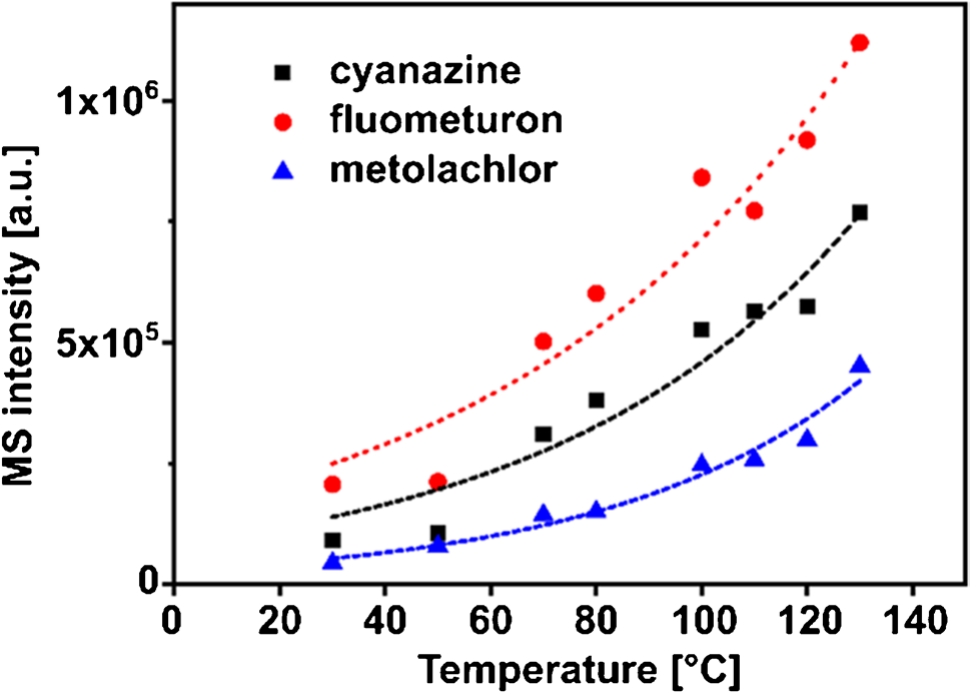
Dependency between column temperature and maximal peak intensity of selected analytes, data are withdrawn from separations illustrated in Fig. 2A

Based on the recorded chromatograms, selected parameters such as retention time, peak width, and maximum peak intensity of the separated compounds were analyzed to evaluate the impact of the different isothermal column conditions. Before discussing each parameter individually, it is worth noting that increased column temperature had a positive effect on all of the parameters mentioned. For example, the total run time of recorded chromatograms was dramatically reduced from over 5 min at 30°C to less than 20 s at 130°C, with retention time reproducibility of 0.7% for selected analytes (*n*=3, retention of metolachlor at 70°C, illustrated in Electronic Supplementary Material Fig. S5). To continue assessing retention time data on the level of individual compounds, a Van’t Hoff plot was created by plotting the logarithmic separation factor of selected analytes against the reciprocal microcolumn temperature (see Electronic Supplementary Material Fig. S6). The observed linear relationships indicate that increasing temperatures equally affect the retention mechanism of each desired compound. A reduction in peak width accompanies decreased chromatographic run time of known pesticide sample mixture (see Electronic Supplementary Material Fig. S7). This confirms the effect of reduced longitudinal diffusion of the sample, as the residence times on the microcolumn are drastically reduced due to the temperature-induced increase in flow rate. In addition to the reduced retention time and peak width, the maximum MS signal intensity as a function of temperature was investigated as a third parameter from the series of injections under isothermal conditions ranging from 30 to 130°C. For this purpose, the maximum peak intensities of the extracted ion chromatograms of the protonated analyte ion species were retrieved and analyzed and are plotted in Fig. 3.

**Fig. 4:**
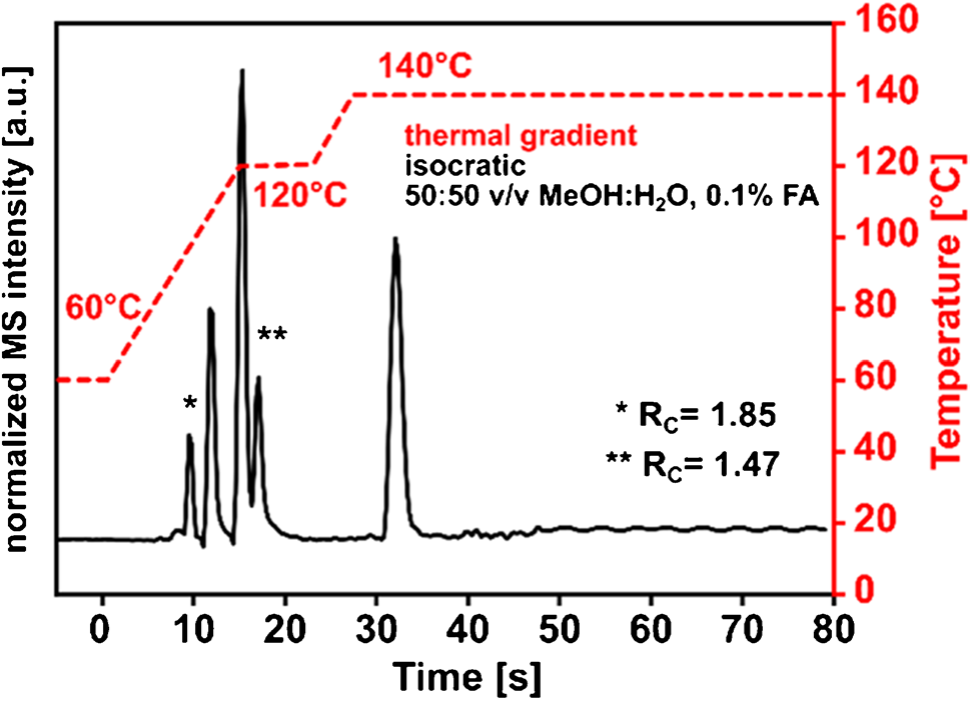
Illustration of a HTchipHPLC ESI MS measurement utilizing a thermal gradient condition. 2-step thermal gradient from 60 to 140°C, column length: 35 mm, material: XBridge C18 BEH, dp=2.5 µm, maximal elution pressure: 133 bar, sample solution was identical to Fig. 2A. R_C_, resolution of the critical peak pair

 There, an analyte-dependent 5 to 10-fold increase in the MS signal is observed for cyanazine, fluometuron, and metolachlor when the column temperature is increased from 30 °C to 130 °C. Since temperature reduces the surface tension of the methanol-water eluent used, the desolvation process is improved during electrospray ionization. Studies investigating ESI signal intensities using non-miniaturized columns reported a 1.7-fold when column temperatures were raised to 150°C [31]. When evaluating the chromatograms of the isothermal separations of the pesticide mixture, it is noticeable that the early eluting hydrophilic compounds have insufficient resolution for separations above the 70°C mark. Since the developed HTchipHPLC MS system allows rapid temperature adjustment, a thermal gradient can be applied to improve the separation. A two-step temperature program was started simultaneously with sample elution, raising the column temperature from 60 to 140°C within 30 s. This resulted in a total analysis time of only 36 s. Compared to an isothermal operation, the thermal gradient significantly improved resolution and peak shape, as can be seen in the example of two selected critical peak pairs (*R_C_, **R_C_) in Fig. 4.

For completeness, a comparative illustration between a solvent and thermal gradient can be found in Electronic Supplementary Material Fig. S8 and Tab. S1. Like other chemical research areas, sustainability awareness is becoming increasingly crucial in separation sciences. Therefore, the so-called green chromatography is currently a highly active field of research [40, 41]. An essential aspect of greening liquid chromatography involves the employment of environmentally less harmful eluents, such as ethanol. It is much easier to replace conventional methanol-based eluents with ethanol-based eluents in HTchipHPLC as viscosity and column backpressure are reduced by utilizing higher temperatures. The effect of column back pressure decrease of the used HT-HPLC chip is displayed in Electronic Supplementary Material Fig. S9.

**Fig. 5:**
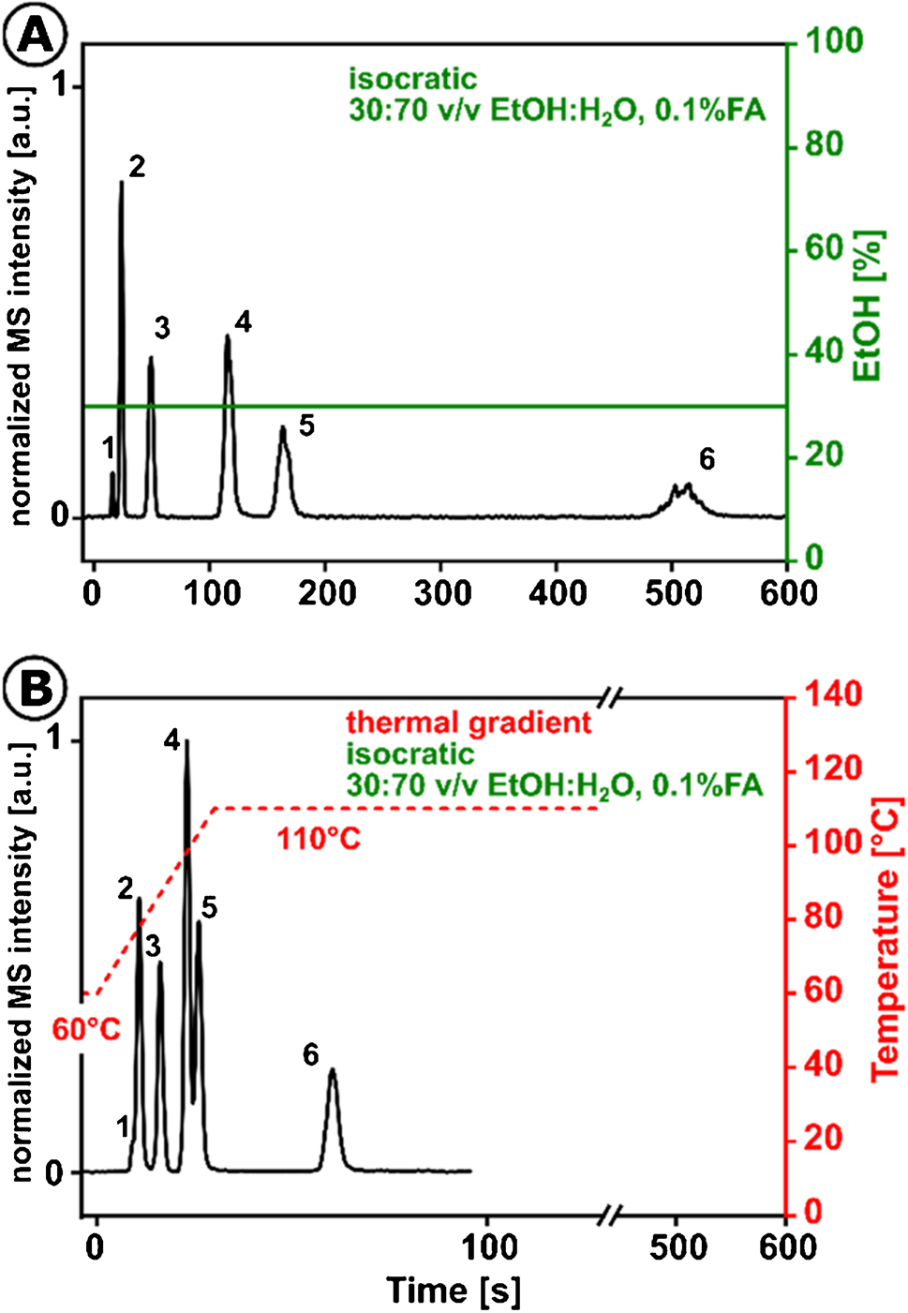
Greening of HTchipHPLC ESI MS illustrated by **A** isocratic separation using 30% v/v EtOH:H_2_O, both 0.1% FA under ambient conditions, linear velocity 2.21 mm/s, *H*=50934 plates m^−1^ and **B** thermal gradient 60 to 110°C under isocratic conditions 30% v/v EtOH:H_2_O, 0.1% FA, linear velocity 3.84 mm/s, H=54523 plates m^−1^, column length 35 mm, material C18 BEH XBridge, dp=2.5 µm, sample fenuron (2, *c*=20 µM), diuron (3, *c*=70 µM), fluometuron (4, *c*=50 µM), tebuthiuron (5, 50 µM), metolachlor (6, 100 µM), and thiourea (1, 5 mM) as deadtime marker in 40:60 MeOH:H_2_O, elution pressure: 133 bar

The results of the successful usage of an ethanol-based eluent are shown in Fig. 5. A detailed version can be seen in Electronic Supplementary Material Fig. S10, and Tab. S2. There, a pesticide mixture is separated with a binary ethanol-water eluent at a temperature gradient of 60 to 110 °C to accelerate chip chromatography under ambient conditions from over 500 s to 60 s. It should be pointed out that compared to methanol, ethanol’s stronger eluotropic strength reduces the organic modifier’s volume fraction down to 30%. As the higher surface tension of the ethanol-containing eluent and the assumed phase transition make it difficult to form an electrospray, the column temperature is limited to 110°C [42].

Since phase transition must be avoided to accelerate high-temperature chip chromatography MS further, prospective developments should focus on integrating pressure-controlling elements in the post-column region. A restrictive micro-channel connecting column and emitter manufactured by state-of-the-art micro-machining or implementing a microfluidic pressure regulator are promising options [43–45].

## Conclusion

This study demonstrates the first successful coupling between high-temperature chip-based HPLC and ESI-MS. The developed HTchipHPLC ESI MS system could operate reliably isothermally until 130°C. This facilitated high-speed chip separations with run times of a few seconds and increased peak intensity compared to ambient conditions.

Due to the low thermal mass of the HT-HPLC chip and the associated rapid heat exchange, it was possible to apply a thermal gradient using environmentally friendly ethanol-water eluents as an alternative to the solvent gradient.

The low microliter system volume of the HT-HPLC chip and the use of temperature as an external, easy-to-use elution control parameter provide an ideal combination to push cycle times in ultrafast chromatography further into the range of a few seconds and below.

### Supplementary Information

Below is the link to the electronic supplementary material.

Supplementary file1 (DOCX 1900 KB)
