# Supplementary material for: High-temperature ultrafast ChipHPLC-MS
Source: Anal Bioanal Chem. 2023 Dec 19;416(4):1023–31. doi: 10.1007/s00216-023-05092-w (PMC10800301; doi:10.1007/s00216-023-05092-w)
Supplement: Supplementary file 1 — Supplementary file1 (DOCX 1900 KB) [file 216_2023_5092_MOESM1_ESM.docx]

**Supporting information**

High-Temperature Ultrafast ChipHPLC-MS

Chris Weise, Hannes Westphal, Rico Warias, and Detlev Belder*

*Institute of Analytical Chemistry, University Leipzig, Linnéstrasse 3, 04103 Leipzig, Germany*

Corresponding author:

*Email Detlev Belder: belder@uni-leipzig.de

**Table of content**

Fig. S1 – Detailed instrumental setup for HTchipHPLC MS page 2

Fig. S2 – Pressure curve during on-column injection cycle page 3

Fig. S3 – Preliminary experiment page 4-5

Figs S4 – Recorded ESI MS spectra page 6

Fig. S5 – Reproducibility at isothermal operation 70°C page 7

Fig. S6 – Van't Hoff plot of isothermal separations page 8

Fig. S7 – Peak width at different column temperatures page 9

Fig. S8 – Comparative between solvent gradient and temperature gradient page 10

Tab. S1 – Chromatographic parameters from solvent- and thermal-based

gradient elution page 11

Fig. S9 – Temperature dependency of maximum elution pressure page 12

Fig. S10 – Greening of HTchipHPLC MS page 13

Tab. S2 – Comparison of chromatographic parameters from green separations page 14

– References page 14

Fig. S1 – Detailed instrumental setup used for HTchipHPLC MS

**
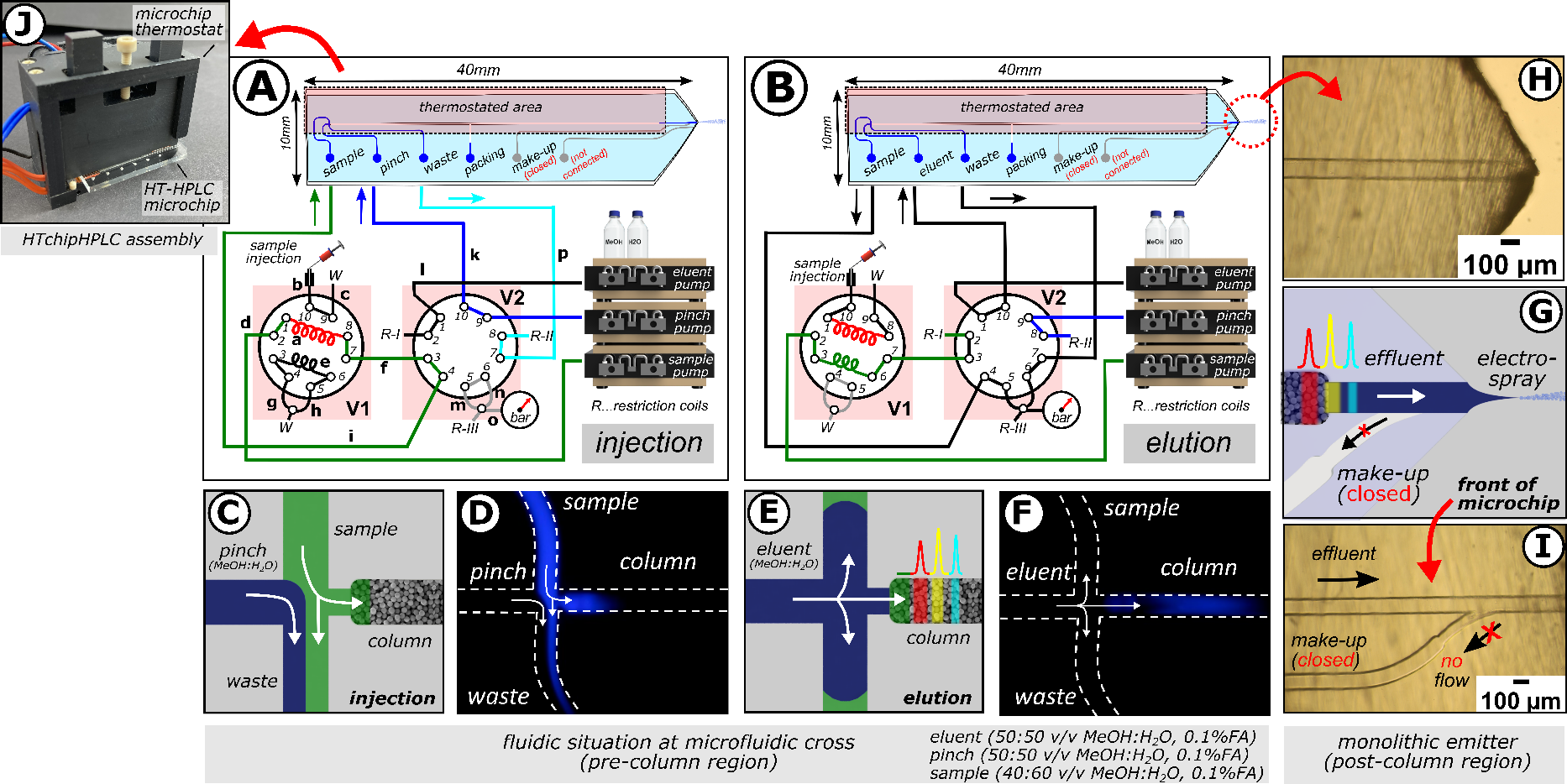
**

**Fig. S1** – Detailed representation of the instrumental setup for HTchipHPLC MS with the corresponding illustration of the on-chip fluidics during (A) injection and (B) elution. Flow paths of the sample stream (green), pinch stream (blue), and eluent stream (black) are colored. Since the sample and pinch streams leave the chip at the waste outlet, the corresponding tubing is colored cyan. In this fluidic situation, capillary tubings not connected to a pumping device are colored in grey. In addition, arrows indicate the direction of flow. Insights of these situations at the microfluidic injection cross are illustrated schematically in (C) for the injection and in (E) for elution and by injections of a fluorescent sample (100µM c120 in MeOH) in (D) for injection, and (F) for elution. (G) Illustration of the post-column region with photographic insight of the (G) emitter tip and the (I) post-column junction. (J) Photographic image of the microchip thermostat used during the study. An overview of the capillaries used within the setup is listed here: (a) - ID 125µm, 40cm, (b) - ID 75µm, 30cm, (c) - ID 125µm, 15cm, (d) - ID 75µm, 30cm, (e) - ID 125µm, 15cm, (f) - ID 50µm, 20cm, (g) - ID 125µm, 15cm, (h) - ID 125µm, 15cm, (i) - ID 75µm, 30cm, (j) - ID 75µm, 30cm, (k) - ID 75µm, 30cm, (l) - ID 75µm, 30cm, (m) - ID 75µm, 15cm, (n) - ID 75µm, 15cm, (o) - ID 100µm, 10cm, (p) - ID 75µm, 30cm, (R-I) - ID 50µm, 180cm, (R-II) - ID 50µm, 30cm, (R-III) - ID 50µm, 180cm, OD 360µm if not note otherwise, A pressure sensor (Inline pressure sensor, Duratec, Germany) used for pressure observations during elution mode.

The presented Fig. S1 shows a detailed instrumental setup required to perform HTchipHPLC MS. It includes the HT-HPLC microchip and its features for MS coupling, the external fluidics, and two switching valves, which are needed to enable the on-column injection principle. The on-column injection principle is based on two flow situations at the microfluidic injection cross, injection and elution, shown in Fig. S1, labeled as C and E, respectively.

In injection mode (Fig. S1 A, C, and D), a sample plug is pumped from the sample loop (approx. 5µL) to the on-chip injection cross. At the injection cross, the sample plug stream meets the pinch stream and undergoes flow splitting according to the ratio determined by the restrictions attached to the injection cross. During this procedure, a part of the sample plug flows directly to the column and accumulates at the column head. In contrast, the remaining sample stream fraction flows laminarly along with the pinch stream into the waste and is discarded. To avoid phase separation during sample injection under higher temperature conditions, a backpressure of 8 bar is established by the joined streams of the sample and pinch pumps (each at 10µL/min) and a capillary restriction (R-II in Fig. S1).

Elution mode starts by changing the valve V2 configuration from position 1-2 to 1-10 (Fig- S1 B, E, and F). The sample and pinch stream are decoupled during the elution mode from flowing on the chip. Instead, a high eluent flow accesses the microchip and gets split between the sample line, waste line, and column. Due to the capillary restriction in the sample and waste line (R-III in Fig. S1), the high eluent flow rate leads to a rapid pressure increase at the injection cross and initiates sample elution. Exemplarily, this behavior is illustrated by the pressure curve in Fig. S2. Herein, a maximal elution pressure of 133bar for an on-column injection using a 30:70 v/v EtOH:H_2_O, 0.1%FA eluent at a column temperature of 100°C was achieved. The time scale of the diagram has a negative offset to ensure a reproducible starting point of HTchipHPLC, even if the injection times need to be adjusted. During the study, an injection time of 15 seconds was used for all measurements. This was executed by a semi-automated injection protocol using Clarity Software Package (Data Apex, Czech Republic). During all measurements, the inlet port for dosing a post-column make-up solvent was closed off to prevent flow splitting into the make-up channel and, therefore, sample loss during operation (Fig. S1-I).


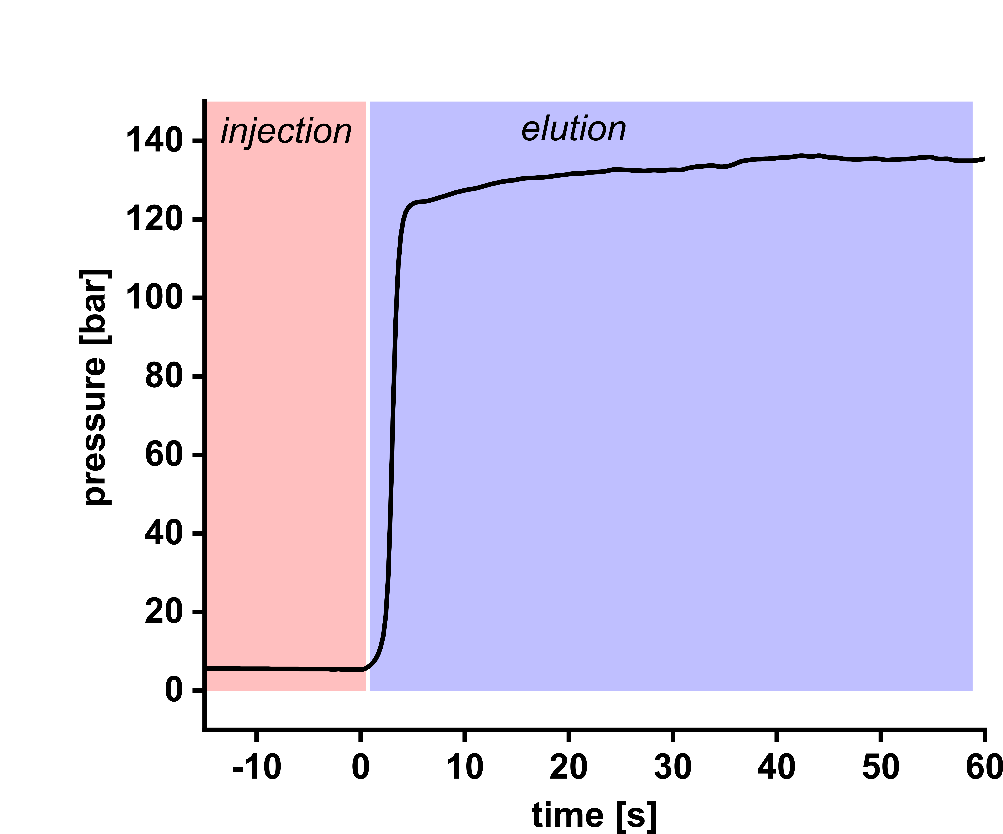


**Fig. S2** – Pressure curve during on-column injection cycle applied for HTchipHPLC ESI MS operation, eluent: 30:70 v/v EtOH:H_2_O, 0.1%FA at microcolumn temperatures of 100°C. A negative offset of 15 seconds (corresponds to the injection time) was introduced to obtain a reproducible starting point of HTchipHPLC

Fig. S3 – Preliminary experiment
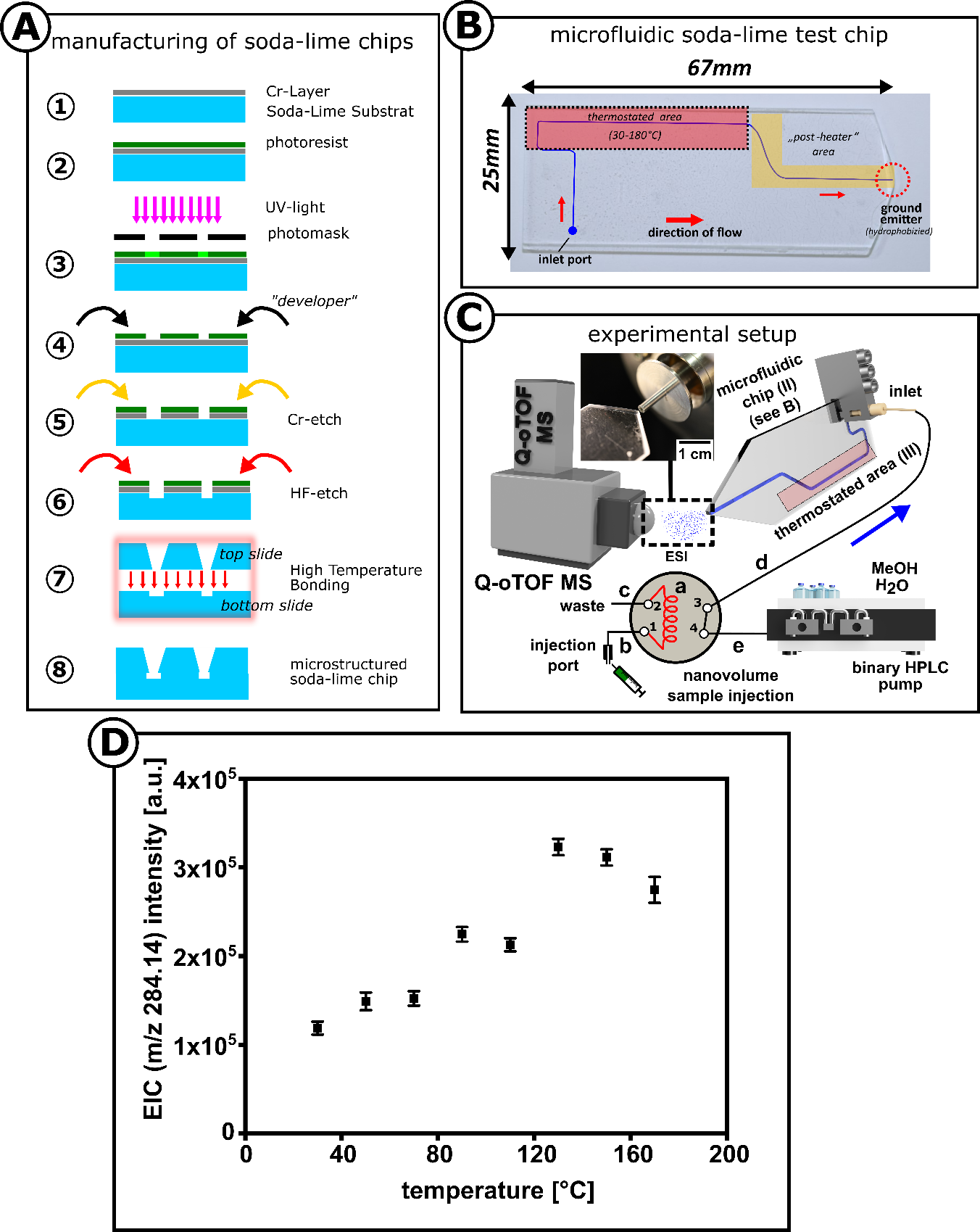


**Fig. S3** – (A) Overview of the soda-lime chip manufacturing process (B) Photography of microchip used for preliminary experiments - The microchip was made of two fusion-bonded soda-lime glass slides (25x75x1mm). The bottom slide contained a single chip channel (trapezoidal channel profile, 110µm in width, 35µm in depth) fabricated using photolithography and HF-wet-etching. The top slide had a powder-blasted, conical inlet port. During the preliminary experiment, the microchip was partially heated by the thermostat (the heated area is illustrated in red). The chip area downstream of the heated region allowed heat exchange and was labeled as a post-heater area and marked in orange. A ground chip emitter was used for MS coupling. (**C**) Schematic drawing of the fluidic circuitry involved in external sample injection on a thermostated microchip (a) – internal loop (V=20nL), (b) ID 100µm, 10cm (c) - ID 125µm, 20cm, (d) – ID 75µm, 15cm (e) - ID 125µm, 35cm + OD 1/16, 120µm, 50cm, all capillaries are made of PEEK and have an OD of 360µm, if not mentioned otherwise, (D)Temperature impact on signal intensity of protonated metolachlor ions (m/z 284.14), measurements made in triplicates

The impact of temperature on MS Signal was investigated during the preliminary experiment. Therefore, a simplified microfluidic chip system without a chromatographic column was fabricated according to the published rapid prototyping protocol [1].

A schematic overview is illustrated in S3A. Briefly, a chrome-sputtered microscopic slide made of soda-lime glass (76mm x 26mm, Thermo Fisher, USA) was spin-coated (30s at 3000rpm) with a positive UV-sensitive photoresist (AZ1518, Microchemicals, Germany). After thermal back out (10 mins at 110°C), the spin-coated slide was covered with a photomask, with the final microfluidic design printed to it (using M3 foil), and exposed to UV light for 30 seconds. Subsequently, the developer solution removes the UV-exposed parts of the photoresist (AZ351B, Microchemicals, Germany). Next, the slide underwent chrome-etching for 1min using a Cr01 etching solution (TechniEtch, Germany) and HF etching using a buffered hydrofluoric acid (BOE 7:1, Microchemicals, Germany) for 50mins. The etching took place in a dedicated teflon container under constant stirring. Once the etching procedure was finished, the remaining photoresist and chrome were removed from the surface of the bottom slide of the prospective microchip. Inlets were powder blasted into the top slide to allow fluidic access. Prior the bonding process both microscopic slides underwent an activation/cleaning step (1h submersed in an aqueous H_2_O_2_-NH_3_ solution) and were placed into a furnace (P330, Nabertherm, Germany). The following high-temperature bonding program was applied (30min – ramp to 500°C and hold it for 30mins – 550°C for 30mins – 650°C for 3h – cool down in 8h) to create the pressure-tight microfluidic soda chip used in the study.

The soda-lime glass microchip (Fig. S3 B) contains a single channel leading into a monolithic emitter. The emitter was ground and hydrophobized analogously to the borosilicate chip employed for the chromatographic experiments. For the experimental part, the described chip was connected to a fluidic circuitry setup (Fig. S3 C), and metolachlor sample (c=150µM dissolved within a 50:50 v/v MeOH/water 0.1% formic acid) was injected via an external nano volume injection valve (20nL injected volume, Cheminert, VICI, Switzerland). The injected sample was pumped by an isocratic HPLC pump (1260 Infinity, Agilent Technologies, USA) operating at 70:30 v/v methanol/water containing 0.1% formic acid at a flow rate of 1µL/min onto the microchip. The world-to-chip transfer of the fluidics was ensured by connecting metal clamps to the inlet port.

Results from this preliminary experiment are illustrated in Fig. S3D. Therein, maximal intensities of the extracted ion chromatogram of the protonated metolachlor ions (m/z 284.14) at microchip temperatures, ranging from 30 to 180°C are displayed. The data show that MS signals can be detected even at applied temperatures above the boiling point of the eluents. Maximum MS signal intensities increase to 130°C, highlighting the positive effects of a pre-heated liquid for analyte detection in MS interfacing.

Fig. S4 – Recorded Mass Spectra of analytes


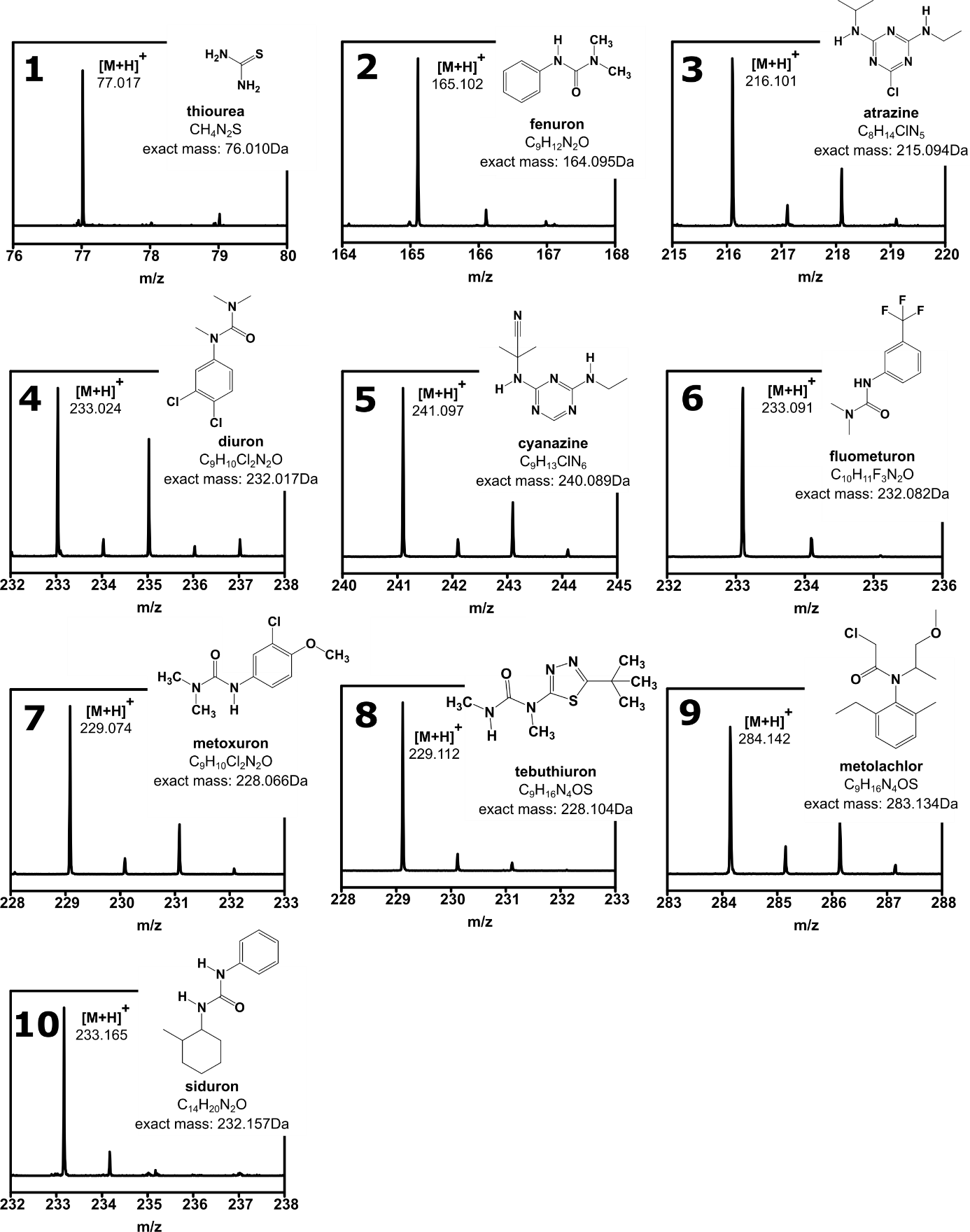


**Fig. S4** – Recorded Q-TOF spectra of separated analytes acquired after performing HTchipHPLC, (1) thiourea, (2) fenuron, (3) atrazine, (4) diuron, (5) cyanazine, (6) fluometuron, (7) metoxuron, (8) tebuthiuron, (9) metolachlor, (10) siduron all detected as [M+H]+, unprotonated structure, sum formula, exact masses (Da) are displayed.

Fig. S5 – Peak properties and their reproducibility

To highlight the reproducibility of the developed HTchipHPLC MS setup, three consecutive injections of a pesticide mixture at isothermal column conditions of 70°C were conducted. The evaluation of the chromatograms of the injection series showed excellent reproducibility of retention time and peak width (figure S5-A). The late eluting metolachlor peak was a subject of investigation regarding the peak shape, which proved to be gaussian-like (Fig. S5-B). Tab.1 summarizes the peak properties of the analytes separated in Fig. S5A. The reproducibility of the different peak features (retention time, peak area, and resolution) under three different isothermal conditions are shown exemplarily for metolachlor (Tab.2).


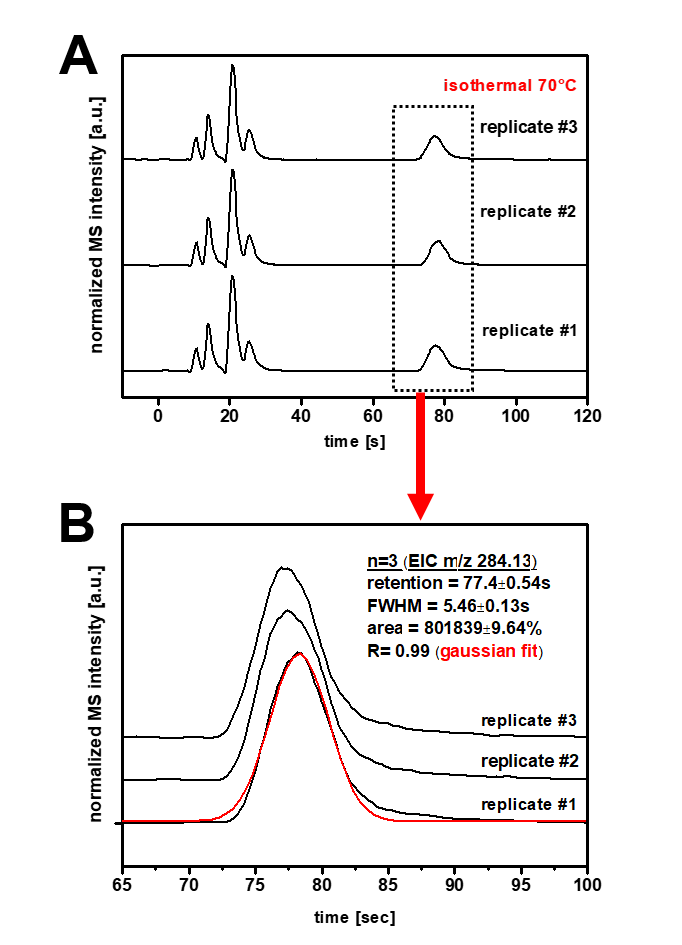


**Fig. S5** - Reproducibility of HTchipHPLC MS system using the example of an isothermal separation under isocratic conditions (n=3, 50:50 v/v MeOH:H_2_O at 70°C, C18 BEH XBridge, dp=2.5µm), (A) HTchipHPLC MS chromatograms of recorded replicates, (B) insights of the late eluting peak of the chromatograms obtained by HTchipHPLC MS from a sample mixture composed of fenuron, cyanazine, diuron, fluometuron and metalochlor all 50µM in 40:60 v/v MeOH:H_2_O, t_injection_=15sec

Fig. S6 – Van't Hoff plot to illustrate the impact of temperature on separation factor

When the logarithmic separation factors of the individual analytes, which are calculated from the retention times retrieved from the isothermal separations, are plotted against the applied microcolumn temperature, a linear relationship of all selected analytes (R^2^ > 0.98) can be observed (Fig. S7). This points out that the present retention mechanism for the selected analytes is equally affected by the rising temperature, and no interferences, which can be related to phase transition or pH effects under higher temperatures, are present [2, 3]. Since no dead time marker was used by the time, fenuron, the first eluting compound of the pesticide mixture, was used as a “pseudo”-dead time marker for calculations. As fenuron slightly interacts with the column, a small offset is introduced. For upcoming separations, thiourea was presented at a dead time marker.


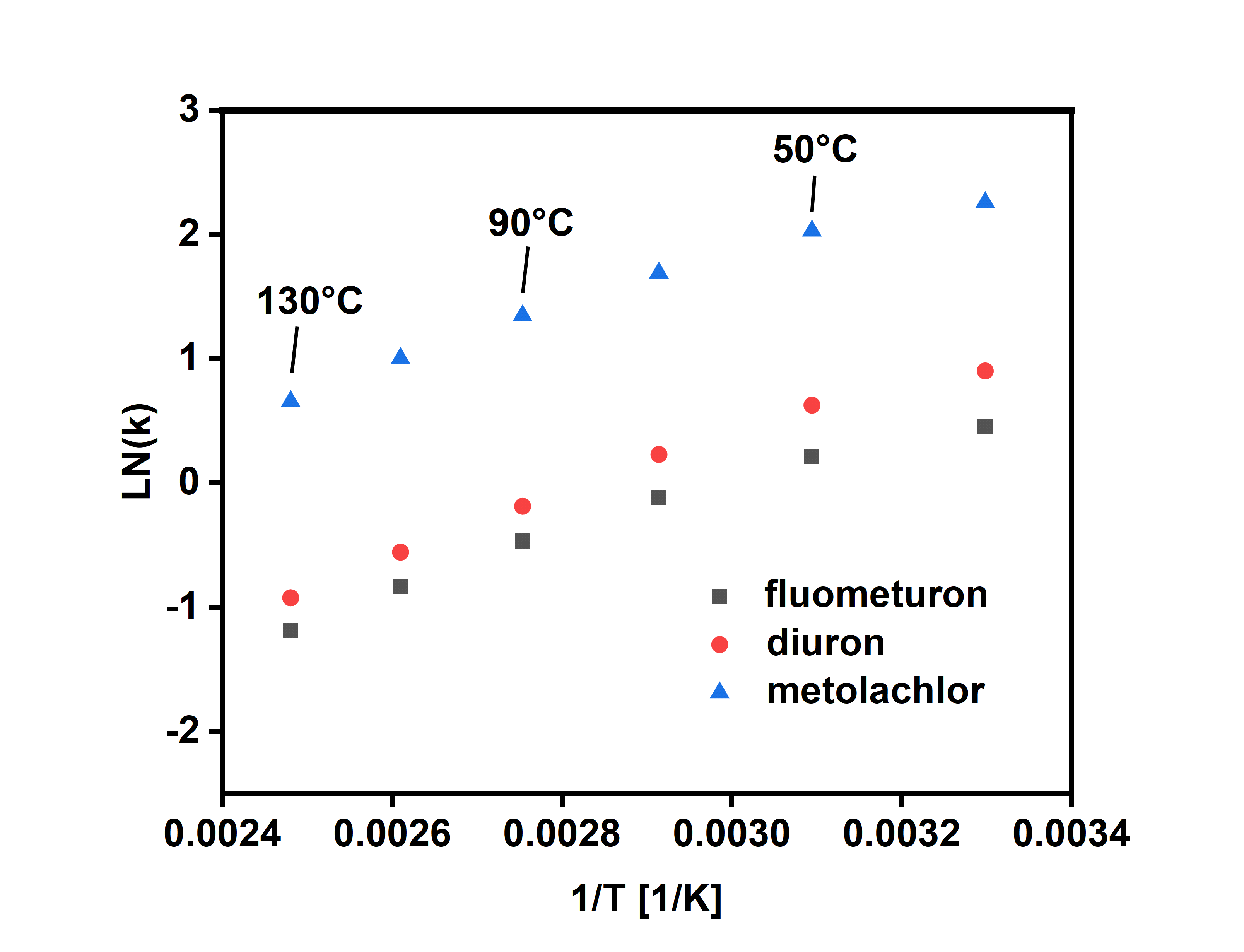


**Fig. S6** - Van't Hoff plot to illustrate temperature dependence of retention - corresponding data from **Fig.2A** in the manuscript

Fig. S7 – Peak width at different column temperatures

As described in the main paper, reduced longitudinal sample diffusion is caused by reduced residence times on the microcolumn due to the temperature-induced increase in flow rate leading to decreasing peak widths. In the manuscript, the peak widths' behavior as a function of temperature is only briefly discussed. The apparent tendency to reduced peak widths as a function of microcolumn temperature for selected analytes is illustrated in Fig. S7.
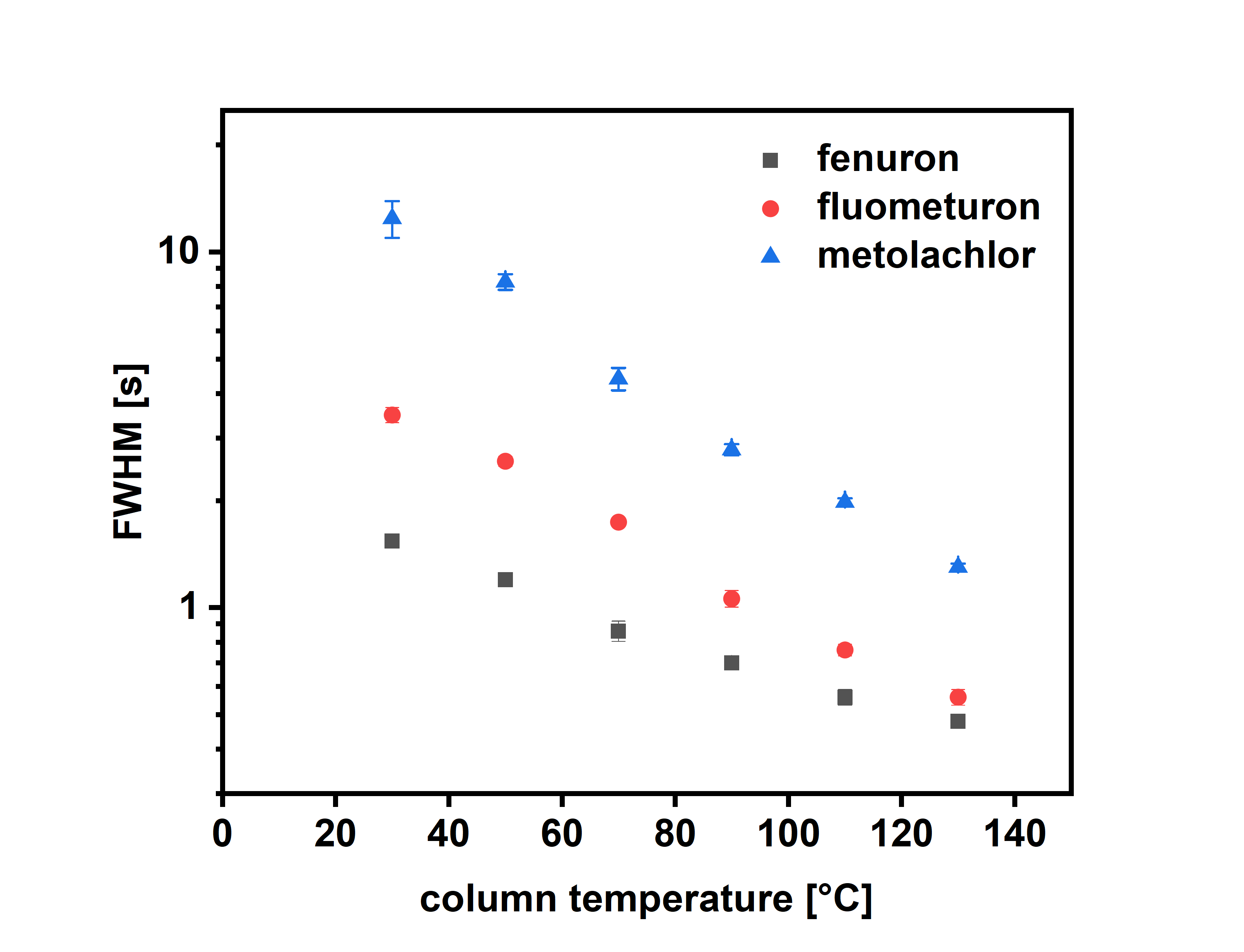
**Fig. S7** - Peak width at different column temperatures - Data are retrieved from the reversed-phase separation under isothermal conditions (from ϑµ-column =30°C to ϑµ-column =130°C) with ESI-MS detection (figure 2A in main paper), eluent: 50/50 v/v MeOH/H2O, 0.1% FA, column length 35mm, material XBridge C18 BEH, dp=2.5µm, tinjection=15s, pesticide mixture dissolved in 40/60 v/v MeOH/H2O as described earlier, for better illustration a logarithmic y-axis was chosen.

Fig. S8 - Comparison between solvent gradient and temperature gradient

Since temperature increases the eluotropic strength of common reversed-phase eluents, such as methanol, it can be used for elution control instead of a solvent gradient. For illustration, chip-based separations of a pesticide mixture are compared using a solvent gradient and a thermal gradient for elution (Fig. S8). Thermal gradient resulted in reduced analysis times. Although co-elution is present, peak shapes are sharper, and peak widths are smaller than a solvent gradient. A detailed overview of the chromatographic parameters can be seen in Tab. S1. Since the thermal gradient does not require knowledge about the gradient delay volume prior to separation, its implementation is much more convenient than a solvent gradient. Furthermore, it lowers the organic solvent consumption per analysis and reduces the equilibration times between the runs.


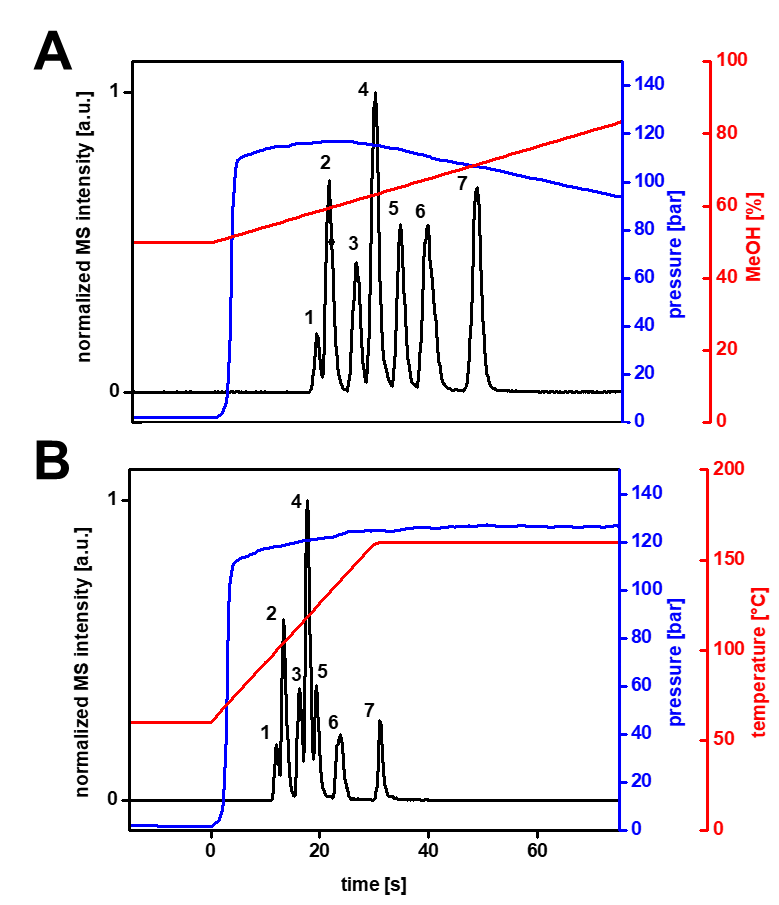


**Fig. S8** – Comparative illustration between solvent gradient and temperature gradient – (A) solvent gradient 50% to 90% v/v MeOH in 80s, linear velocity 1.84mm⋅s-1, H=118071plates⋅m-1 (B) temperature gradient 60-160°C under isocratic conditions 50:50 v/v MeOH:H2O, linear velocity 2.9 mm⋅s-1, H=202683plates⋅m-1, both 0.1% FA, C18 BEH XBridge, dp=2.5µm, sample fenuron (1, 25µM), diuron (2, 50µM), fluometuron (3, 50µM), metoxuron (4, 25µM), tebuthiuron (5, 50µM), siduron (6, 50µM) and metolachlor (7, 50µM) in 40:60 v/v MeOH:H2O, tinjection=15s. Linear velocity was calculated based on the dead marker thiourea. Theoretical plate number H was determined based on retention time and resolution of late-eluting metolachlor.

**Tab. S1** – Comparison of chromatographic parameters resulting from solvent gradient elution and thermal gradient elution

| **analyte** | **retention [s]** | | **peak width [s]** | | **N [plates/m]** | |
| --- | --- | --- | --- | --- | --- | --- |
|  | **solvent gradient** | **thermal gradient** | **solvent gradient** | **thermal gradient** | **solvent gradient** | **thermal gradient** |
| **1** | dead time marker | | | | | |
| **2** | 21.26 | 13.40 | 1.47 | 0.93 | 33108 | 32871 |
| **3** | 26.24 | 16.36 | 1.60 | 1.05 | 42573 | 38707 |
| **4** | 29.65 | 17.78 | 1.44 | 0.81 | 66828 | 76250 |
| **5** | 34.38 | 19.37 | 1.51 | 1.03 | 82059 | 55865 |
| **6** | 39.40 | 23.69 | 2.26 | 1.44 | 48103 | 43139 |
| **7** | 48.34 | 31.13 | 1.77 | 0.87 | 118071 | 202683 |

Fig. S9 – Temperature dependency of maximum elution pressure

The HTchipHPLC MS system can reduce the mobile phase's viscosity to achieve higher linear velocities at lower elution pressure. Fig. S9 illustrates that the reduction in elution pressure of a 50:50 (v/v) MeOH:H_2_O, 0.1% FA at 70°C becomes more pronounced with increasing linear velocity. A high-speed operation under reduced elution pressure is highly attractive for employing greener eluent in HTchipHPLC, as highlighted in Fig. S10.


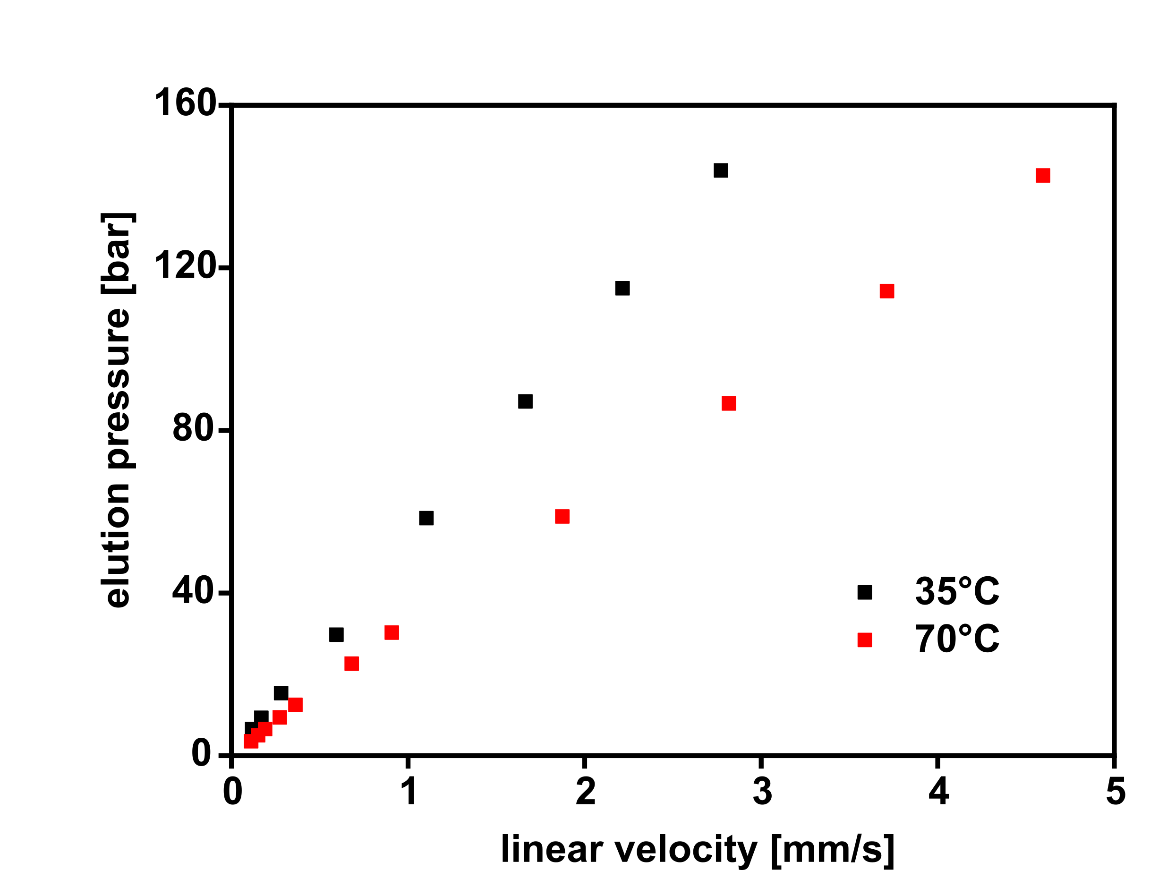


**Fig. S9** - Comparison of maximum elution pressure at different column temperatures, data were generated based on isothermal separation (35°C and 70°C) under isocratic conditions (50:50 (v/v) MeOH:H_2_O, 0.1% FA, sample composed of thiourea (dead time marker, c=5mM), metoxuron (c=50µM), fluometuron (c=50µM) in 40:60 MeOH:H_2_O, C18 BEH XBridge, dp=2.5µm, t_injection_ = 15sec

Fig. S10 – Greening of HTchipHPLC MS

Due to the reduced viscosity, HTchipHPLC allows the substitution of methanol-based eluents with high-viscosity ethanol-based eluents, resulting in the greening of the chipHPLC. To test this relationship, a pesticide mixture was loaded onto the chip-based column and eluted isocratically with a 30:70 v/v EtOH:H_2_O (Fig. S10-A), isothermal at 100°C (Fig. S10-B) and with a thermal gradient (Fig. S10-C). Using temperature, the analysis time could be reduced from over 500 seconds to less than 70 seconds. Furthermore, improved peak widths were observed for the thermal gradient. A detailed overview of the chromatographic parameters can be found in Tab.S2.


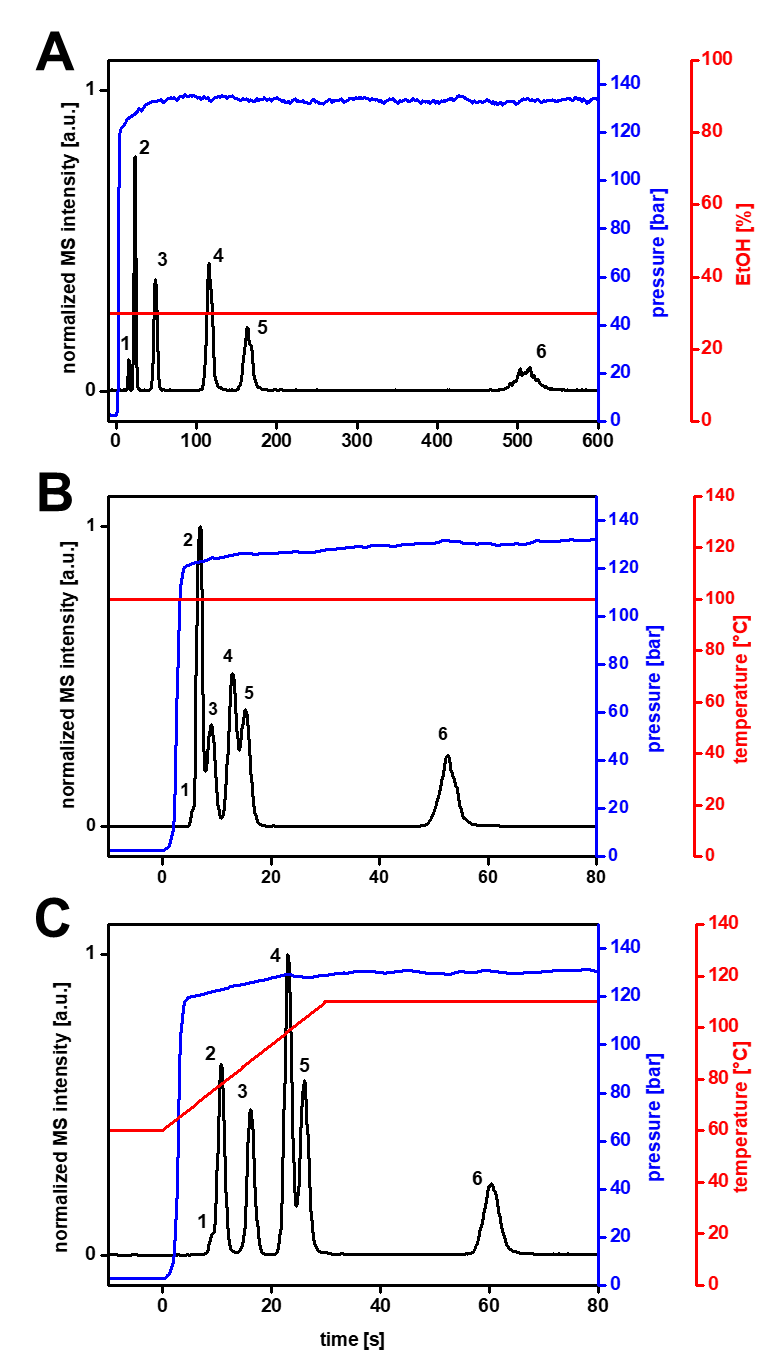


**Fig. S10** - Greening of HTchipHPLC MS – (A) isocratic separation using 30% v/v EtOH:H_2_O, both 0.1%FA, linear velocity 2.21mm⋅s^-1^; H=50934plates⋅m^-1^ (B) isothermal separation at 100°C, linear velocity 6.01mm⋅s^-1^, H=38813plates⋅m^-1^ (C) thermal gradient 60°C to 110°C under isocratic conditions 30% v/v EtOH:H_2_O, 0.1%FA, linear velocity 3.84mm⋅s^-1^, H=54523plates⋅m^-1^, C18 BEH XBridge, dp=2.5µm, sample fenuron (2, c=20µM), diuron (3, c=70µM), fluometuron (4, c=50µM), tebuthiuron (5, 50µM), metolachlor (6, 100µM) and thiourea (1, 5mM) as deadtime marker in 40:60 MeOH:H_2_O, t_injection_=15sec

**Tab. S2** - Comparison of chromatographic parameters resulting from green separations using an isocratic elution (30:70 v/v EtOH:H_2_O, 0.1%FA), isothermal elution (100°C) and thermal gradient (60-110°C)

| **analyte** | **retention [s]** | | | **peak width [s]** | | | **N [plates/m]** | | |
| --- | --- | --- | --- | --- | --- | --- | --- | --- | --- |
|  | **isocratic elution** | **iso- thermal** | **thermal gradient** | **isocratic elution** | **iso- thermal** | **thermal gradient** | **isocratic elution** | **iso-thermal** | **thermal gradient** |
| **1** | deadtime marker | | | | | | | | |
| **2** | 23.53 | 6.82 | 10.77 | 2.78 | 1.15 | 1.38 | 11312 | 5564 | 9580 |
| **3** | 49.05 | 8.86 | 16.19 | 4.78 | 1.89 | 1.46 | 16663 | 3482 | 19411 |
| **4** | 116.35 | 12.99 | 23.10 | 7.83 | 2.00 | 1.52 | 34960 | 6681 | 36699 |
| **5** | 164.17 | 15.15 | 26.01 | 11.13 | 2.23 | 1.88 | 34464 | 7328 | 30300 |
| **6** | 510.70 | 52.60 | 60.43 | 28.47 | 3.36 | 3.26 | 50934 | 38813 | 54523 |

References

1. Gerhardt RF, Peretzki AJ, Piendl SK, Belder D. Seamless Combination of High-Pressure Chip-HPLC and Droplet Microfluidics on an Integrated Microfluidic Glass Chip. Anal Chem. 2017; https://doi.org/10.1021/acs.analchem.7b04331

2. Heinisch S, Rocca J-L. Sense and nonsense of high-temperature liquid chromatography. J Chromatogr A. 2009; https://doi.org/10.1016/j.chroma.2008.11.048

3. Tanase M, Soare A, David V, Moldoveanu SC. Sources of Nonlinear van't Hoff Temperature Dependence in High-Performance Liquid Chromatography. ACS Omega. 2019; https://doi.org/10.1021/acsomega.9b02689
